# Motor imagery training speeds up gait recovery and decreases the risk of falls in patients submitted to total knee arthroplasty

**DOI:** 10.1038/s41598-020-65820-5

**Published:** 2020-06-02

**Authors:** Laura Zapparoli, Lucia Maria Sacheli, Silvia Seghezzi, Matteo Preti, Elena Stucovitz, Francesco Negrini, Catia Pelosi, Nicola Ursino, Giuseppe Banfi, Eraldo Paulesu

**Affiliations:** 1 Psychology Department and NeuroMI – Milan Center for Neuroscience, University of Milano-Bicocca, Milan, Italy; 2 IRCCS Istituto Ortopedico Galeazzi, Milan, Italy; 3 PhD Program in Neuroscience, School of Medicine and Surgery, University of Milan-Bicocca, Milan, Italy; 4 University Vita e Salute San Raffaele, Milan, Italy

**Keywords:** Clinical trial design, Translational research, Medical research, Cognitive control

## Abstract

With Motor imagery (MI), movements are mentally rehearsed without overt actions; this procedure has been adopted in motor rehabilitation, primarily in brain-damaged patients. Here we rather tested the clinical potentials of MI in purely orthopaedic patients who, by definition, should maximally benefit of mental exercises because of their intact brain. To this end we studied the recovery of gait after total knee arthroplasty and evaluated whether MI combined with physiotherapy could speed up the recovery of gait and even limit the occurrence of future falls. We studied 48 patients at the beginning and by the end of the post-surgery residential rehabilitation program: half of them completed a specific MI training supported by computerized visual stimulation (experimental group); the other half performed a non-motoric cognitive training (control group). All patients also had standard physiotherapy. By the end of the rehabilitation, the experimental group showed a better recovery of gait and active knee flexion-extension movements, and less pain. The number of falls or near falls after surgery was significantly lower in the experimental group. These results show that MI can improve gait abilities and limit future falls in orthopaedic patients, without collateral risks and with limited costs.

## Introduction

Total knee arthroplasty (TKA) is the extreme remedy for severe osteoarthritis^1^. Post-surgery rehabilitation typically consists of physical therapy aimed at mechanically restoring the functionality of the musculoskeletal system and gait abilities, by integrating passive and active limb movements^2^.

However, both osteoarthritis and the immobilization and pain after surgery induce a motor impairment that is also associated with changes in the activation of motor brain circuits. For instance, we previously demonstrated that rhizarthrosis is associated with cognitive and neurofunctional changes in the mental representation of hand movements^3^. Patients with rhizarthrosis were slower than healthy controls both in the motor execution and in the imagination of hands movement and they showed reduced activation in the hand primary motor and dorsal premotor cortex when executing hand movements^3^. We found similar results in patients with knee osteoarthritis, whose gait motor imagery (MI) was impoverished and associated with reduced activation of gait motor brain circuits^4,5^. These findings suggest that training based on MI may complement standard physical therapy after orthopaedic surgery, to restore gait motor representations, speeding up rehabilitation.

MI, a mental state when movements are mentally evoked and rehearsed without the need of overt actions^6^, is becoming the cognitive treatment of choice to complement physical rehabilitation because of its functional equivalence with real movements^7–9^.

MI has been used in motor rehabilitation after stroke^10^, after head trauma^11^ and in movement disorders^12^; surprisingly, no much work has been done on the use of MI for the rehabilitation of gait after elective orthopaedic surgery (i.e., surgery that is scheduled in advance because it does not involve a medical emergency). However, patients with purely orthopaedic problems should maximally benefit of mental exercises based on MI because of their intact brains.

We have already demonstrated that motor simulations involved in MI can ease the recovery of post-immobilization syndromes characteristic of hand surgery with better motion and less pain^13^.

Promising evidence is available also on orthopaedic patients submitted to total hip arthroplasty: in combination with action observation of simple and complex gait patterns, MI training led to positive effects on task-specific outcomes following total hip arthroplasty (e.g., Timed Up-and-Go test, TUG^14^), and fast-paced gait speed in a dual-task context^15^.

For what concerns knee surgery, to date only two studies tested the possible clinical efficacy of MI training in post-surgery rehabilitation during the acute phase^16,17^. They reported encouraging results in terms of isometric knee extension^16^ and perceived pain^17^. Mourzarkel and colleagues^18^ demonstrated the efficacy of MI training also in the chronic post-operative phase (six months after the surgery), for what concerns quadriceps muscle strength of the operated knee, active knee flexion measured with gait analysis and speed in climbing and descending stairs^18^.

However, those studies were based on small samples and they didn’t include a placebo cognitive-based control treatment. Moreover, in no study, the risk of falls, possibly the harshest endpoint of any rehabilitation treatment of gait, was considered.

## Aim of the study

In this randomized placebo-controlled study, we tested the clinical efficacy of a MI training, investigating whether this could speed-up the recovery after elective knee surgery in the post-operative acute phase while keeping relevant clinical effects in the long term. In particular, we were interested in learning whether any amelioration was specific for gait abilities, or whether symptoms such as pain would show improvement as well. To document the effects of MI, we used standard clinical measures (e.g., TUG or functional scales) but also more subtle measures, like computerized measures of gait parameters. Importantly, our experimental MI training and a placebo-control training were added to the conventional gait rehabilitation program. Crucially, we also considered long-term effects of the treatment by recording the number of falls or near falls after, on average, two years from surgery.

## Results

### Time up and go (TUG) and hand-walking tasks (HWT)

#### Within-group comparisons

Within-group analyses showed a significant improvement in the TUG execution in both groups, as confirmed by both the statistical comparison and the minimal detectable change measure (see the details in Table 1). Only the experimental group reported an improvement in the TUG Motor Imagery Quality Index (calculated on the basis of the difference in the timing of TUG motor execution and TUG motor imagery, see the methods section).

**Table 1:** Descriptive statistics of the data collected (mean (SD)) before (T1) and after (T2) the intervention, for both the experimental and the control group. MDC = Minimal Detectable Change.

*Parameter*	Experimental Group				Control Group			
	T1	T2	MDC	Statistics	T1	T2	MDC	Statistics
*TUG (s)*	42.28 (34.3)	18.98 (7.3)	16.24	Wilcoxon W = 300, p < 0.001	42.53 (20.1)	23.05 (9.87)	11.37	Wilcoxon W = 276, p < 0.001
*Imagined TUG (s)*	22.20(14.6)	14.82(6.74)	7.13	Wilcoxon W = 262, p < 0.001	26.84(12.56)	16.62 (8.74)	7.11	Wilcoxon W = 290, p < 0.001
*MIQI TUG*	0.57 (0.32)	0.27 (0.29)	0.18	Wilcoxon W = 281, p < 0.001	0.44 (0.27)	0.4 (0.21)	0.25	Student’s t(23)=0.84, p = 0.41
*HWT (s)*	11.00 (6.85)	9.84 (6.74)	3.88	Student’s t(23)=1.4, p = 0.16	11.87 (6.94)	10.27 (6.99)	3.93	Student’s t(23)=1.9, p = 0.07
*Imagined HWT (s)*	10.44 (7.97)	9.93 (7.4)	4.51	Wilcoxon W = 160, p = 0.79	12.39 (6.23)	11.60 (7.44)	3.52	Wilcoxon W = 198, p = 0.18
*MIQI HWT*	−0.09 (0.3)	−0.006 (0.2)	0.17	Student’s t(23)=1.9, p = 0.07	−0.007 (0.3)	0.07 (0.2)	0.17	Student’s t(23)=1.9, p = 0.06
*VAS (Affected Leg)*	4.65 (2.96)	1.71 (1.73)	1.67	Wilcoxon W = 269, p < 0.001	2.74 (2.1)	2.23 (2.06)	1.19	Wilcoxon W = 176, p = 0.04
*VAS (Non-Affected Leg)*	0.17 (0.52)	0.03 (0.09)	0.29	Student’s t(23)=1.3, p = 0.20	0.02 (0.08)	0.00 (0.00)	0.05	Student’s t(23)=1.2, p = 0.23
*Active ROM (Affected Leg) (°)*	17.11 (8.55)	27.44 (12.25)	4.84	Student’s t(17) = 6.4, p < 0.001	19.69 (9.65)	24.62 (8.72)	5.46	Student’s t(17) = 4.2, p < 0.001
*Active ROM (Non Affected Leg) (°)*	35.94 (7.87)	40.29 (9.08)	4.45	Student’s t(17) = 2.5, p = 0.02	38.58 (10.45)	44.10 (8.10)	5.91	Student’s t(17) = 3.5, p = 0.003
*Double Support Phase (%)*	26.39 (18.37)	16.81 (4.54)	10.39	Wilcoxon W = 25, p = 0.01	25.13 (12.07)	26.88 (15.61)	6.83	Wilcoxon W = 43, p = 0.12
*Stance Phase (Non-Affected Leg, %)*	70.76 (6.39)	67.14 (4.36)	3.62	Student’s t(17) = 2.8, p = 0.01	72.47 (5.60)	68.50 (5.08)	3.17	Student’s t(17) = 5.6, p < 0.001
*Swing Phase (Non-Affected Leg, %)*	28.13 (4.67)	32.92 (4.37)	2.64	Student’s t(17) = 9.2, p < 0.001	27.54 (5.60)	31.50 (5.08)	3.17	Student’s t(17) = 5.6, p < 0.001
*Stance Phase (Affected Leg, %)*	65.29 (3.02)	62.93 (3.83)	1.71	Student’s t(17) = 3.2, p = 0.006	66.33 (4.90)	64.68 (4.36)	2.77	Student’s t(17) = 3.7, p = 0.002
*Swing Phase (Affected Leg, %)*	34.67 (3.01)	36.14 (2.82)	1.70	Student’s t(17) = 2, p = 0.04	33.52 (4.82)	35.33 (4.36)	2.73	Student’s t(17) = 4.1, p < 0.001
*Stride Lenght (Non-Affected Leg, m)*	0.30 (0.11)	0.39 (0.10)	0.06	Student’s t(17) = 4.8, p < 0.001	0.29 (0.14)	0.38 (0.11)	0.08	Student’s t(17) = 3.8, p = 0.002
*Stride Lenght (Affected Leg, m)*	0.35 (0.07)	0.41 (0.07)	0.04	Student’s t(17) = 4.4, p < 0.001	0.36 (0.09)	0.39 (0.11)	0.05	Student’s t(17) = 2.2, p = 0.04
*Gait Cadence (steps/min)*	62.43 (12.07)	72.91 (12.03)	6.83	Student’s t(17) = 5.9, p < 0.001	64.21 (16.17)	74.79 (16.88)	9.15	Student’s t(17) = 4.4, p < 0.001
*Gait Speed (m/s)*	0.36 (0.12)	0.49 (0.14)	0.07	Student’s t(17) = 5.8, p < 0.001	0.37 (0.16)	0.51 (0.10)	0.09	Student’s t(17) = 5.9, p < 0.001
*FIM*	97.79 (8.07)	120.38 (9.19)	4.57	Student’s t(23) = 11.9, p < 0.001	96.79 (10.47)	122.79 (2.47)	5.92	Student’s t(23) = 12.9, p < 0.001
*Barthel Index*	70.13 (11.73)	98.33 (2.68)	6.64	Student’s t(23) = 12.2, p < 0.001	68.87 (14.03)	98.92 (2.43)	7.94	Student’s t(23)=11.4, p < 0.001
*ROM*	86.46 (12.22)	107.92 (5.29)	6.91	Student’s t(23) = 10.4, p < 0.001	86.04 (13.90)	108.21 (5.03)	7.86	Student’s t(23) = 8.6, p < 0.001

No differences were found for the HWT, neither for the executed nor the imagined version.

#### Between-group comparisons

**Figure 1:**
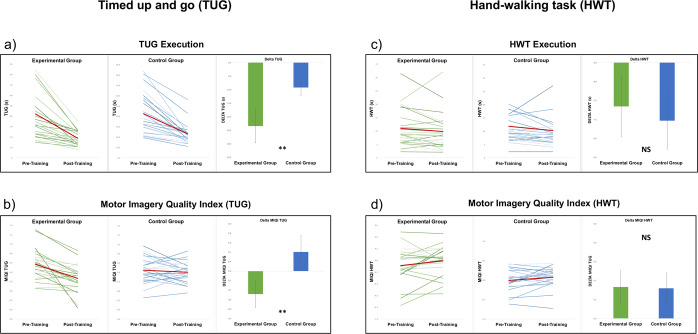
MI training effects on the TUG task (**a**. gait execution; **b**. gait motor imagery, Motor Imagery Quality Index) and on the HWT control test (**c**. hand movement execution; **d**. hand movement imagination, Motor Imagery Quality Index).

Between group-analysis conducted on the delta measure of the time needed for the TUG execution showed a significant effect of Group [t(46) = 2.07, p = 0.04, Cohen’s d = 0.59]: this indicates a greater decrease of the time needed to perform the TUG in the experimental group (see Fig. 1a). The analysis conducted on the TUG Motor Imagery Quality Index (calculated on the basis of the difference in the timing of TUG motor execution and TUG motor imagery, see the methods section) showed an effect of Group as well [t(46) = 3.04, p = 0.004, Cohen’s d = 0.87; see Fig. 1b], indicating a higher improvement of gait MI abilities in the experimental group.

The factor Group was not significant in the analysis conducted on the HWT delta data [t(46) = −0.88, p = 0.38, Cohen’s d = 0.26; see Fig. 1c] and on the HWT Motor Imagery Quality Index (calculated on the basis of the difference in the timing of HWT motor execution and HWT motor imagery, see the methods section) [t(46) = -0.05, p = 0.96, Cohen’s d = 0.01; see Fig. 1d], indicating that the improvement was specific for the motor representations of gait.

### Evaluation of pain

#### Within-group comparisons

Within-group analyses showed a significant decrease in pain perception for the affected knee, only in the experimental group. No changes were found in the control group, neither for the affected nor for the non-affected knee.

#### Between-group comparisons

**Figure 2:**
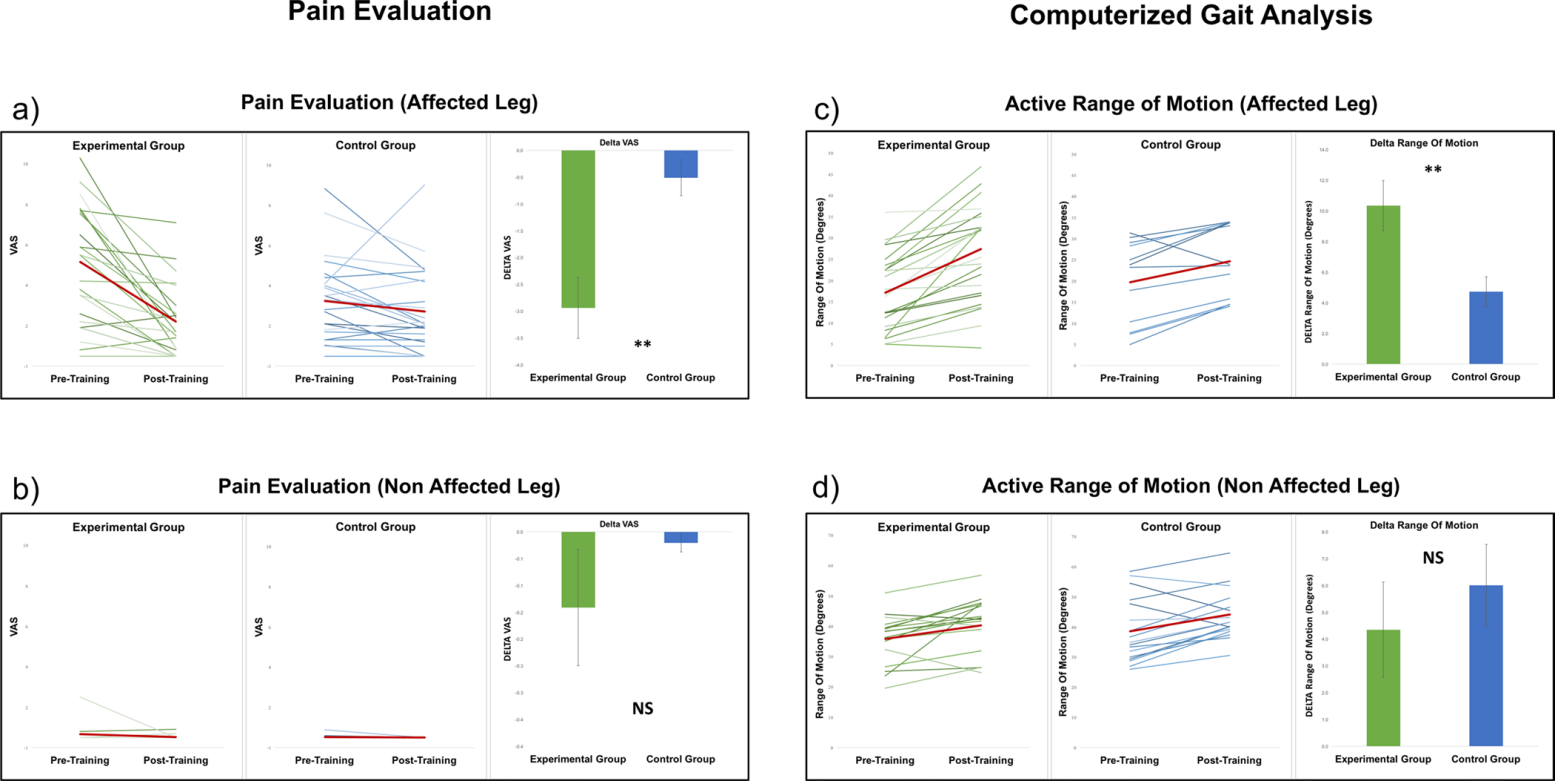
MI training effects on pain perception (**a**. affected leg; **b**. non-affected leg) and on the active range of motion data (**c**. affected leg; **d**. non-affected leg).

The delta measure of pain perception was significantly different between the two groups [Mann-Whitney U = 126, p = 0.002, Cohen’s d = 1.1], indicating a greater decrease in the level of pain in the experimental group (see Fig. 2a). As expected, no differences were found for the non-affected knee [Mann-Whitney U = 268, p = 0.48, Cohen’s d = 0.32, see Fig. 2b].

### Gait analysis

#### Within-group comparisons

Within-group analyses showed a significant improvement for almost all the parameters collected during the computerized gait analyses in both groups, as confirmed by both the statistical comparisons and the minimal detectable change measures (see the details in Table 1).

#### Between-group comparisons

**Figure 3:**
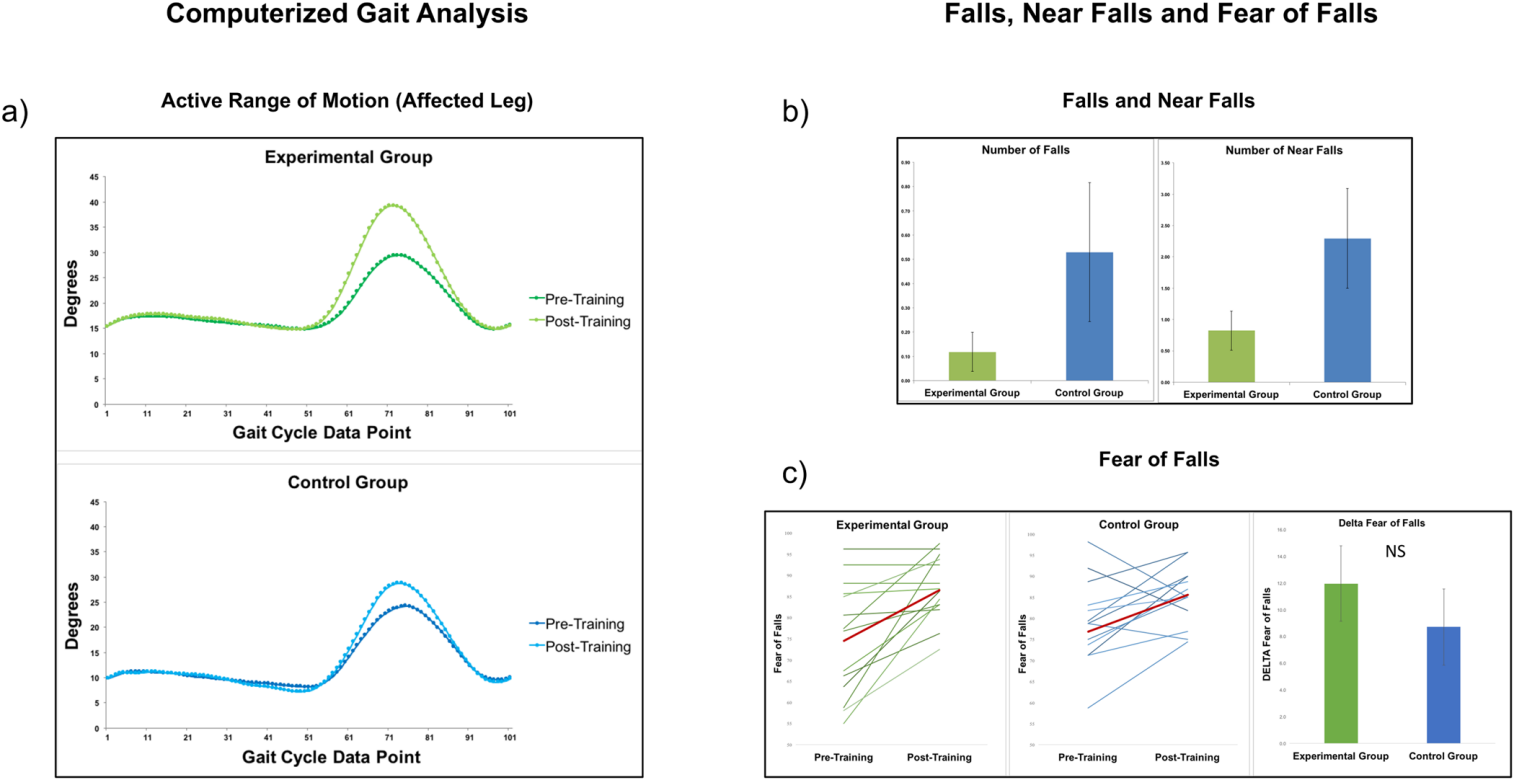
MI training effects (**a**) on active range of motion data, (**b**) on the number of number of falls/near falls and (**c**) on fear of falls.

We found a greater improvement of the active range of motion of the affected knee in the experimental group [t(34) = 2.69; p = 0.01, Cohen’s d = 0.89; see Fig. 2c], which was not significant for the non-affected knee [t(34) = −0.5; p = 0.62, Cohen’s d = 0.16; see Figs. 2d and 3a], as expected.

None of the other temporal and spatial parameters showed significant group differences (see Table 2 for further details).

**Table 2:** Between-group comparisons for the gait analysis data (statistical details).

Parameter	Statistical Test	Statistics	DF	p-value	Cohen’s d
Double Support Phase	Mann-Whitney U	140	—	0.67	−0.55
Stance Phase (Non-Affected Leg)	Mann-Whitney U	140	—	0.49	0.14
Swing Phase (Non-Affected Leg)	Mann-Whitney U	125	—	0.24	0.21
Stance Phase (Affected Leg)	Student’s t	−0.58	34	0.57	−0.19
Swing Phase (Affected Leg)	Student’s t	−0.61	34	0.55	−0.20
Stride Lenght (Non-Affected Leg)	Student’s t	0.34	34	0.73	0.11
Stride Lenght (Affected Leg)	Student’s t	1.34	34	0.19	0.45
Gait Cadence	Student’s t	0.03	34	0.97	0.02
Gait Speed	Student’s t	−0.03	34	0.97	−0.02

### Falls, near falls and fear of falls

The number of falls and near falls was significantly lower in the experimental group, on average, 2 years after surgery, independently from the time passed from the surgery [Factor Group: z = 3.58, p < 0.001, exponential effect size = 1.75, 95% Confidence Intervals [1.31, 2.29], standardized mean difference effect size = 0.96, 95% Confidence Intervals [0.39, 1.72]; interaction Group*Time: z = 1.38, p = 0.17, exponential effect size = 1.33,Confidence Intervals [0.88, 2.01], standardized mean difference effect size = 0.43, 95% Confidence Intervals [−0.15, 1.33]; see Fig. 3b].

We did not find any effect for the fear of fall, as measured by the ABC scale [Mann-Whitney U = 103, p = 0.88, Cohen’s d = 0.02; see Fig. 3c].

### Functional scale and passive range of motion

#### Within-group comparisons

Within-group analyses showed a significant improvement in the functional scales scores and in the passive range of motion in both groups, as confirmed by both the statistical comparisons and the minimal detectable change measures (see the details in Table 1).

#### Between-group comparisons

No significant differences between the two groups were found in the functional scales nor in the passive range of motion (Functional Independence Scale, FIM: t(34) = −1.55, p = 0.13, Cohen’s d = 0.51; Barthel Scale: t(34) = −1.07, p = 0.29, Cohen’s d = 0.36; Passive Range of Motion, affected knee: t(34) = 0.77, p = 0.45, Cohen’s d = 0.25). A supplementary analysis conducted on a subgroup of 16 patients (8 per group) revealed that even the scores obtained in specific gait-related items (i.e., gait and stairs items of the Barthel and the FIM scales) were not significantly different between the two groups (see Supplementary Table S2).

## Discussion

The aim of this study was to test the clinical efficacy of a mental MI training program in orthopaedic patients, by investigating whether this could improve gait abilities and speed-up the recovery after elective knee surgery.

Our study has several strength points: we included a placebo-control training for the control group and we considered as outcome variables both standard clinical measures (e.g., the TUG test, FIM Scale and Barthel Index, passive ROM) and more subtle parameters (e.g., computerized indexes of gait). Finally, we studied and trained our patients in the post-operative acute stage, but we also considered important long-term outcomes, such as the risk for adverse events like falls.

Our results suggest that the motor imagery (MI) training can improve gait abilities and limit new falls in patients with recent total knee arthroplasty. In particular, behavioral and clinical data revealed that the MI training was sufficient to speed up gait recovery, improving functional gait features, reducing the magnitude of pain, and enhancing the ability to efficiently evoke gait motor representations. We start by discussing the effects of MI training on gait motor performance. Next, we consider the impact of the training on measures typically adopted in clinical settings (pain level, functional scales and knee range of motion). We then address the kinematics data collected by means of computerized gait analysis and we conclude with considerations on the impact of our training on the risk of falls. Finally, we discuss the clinical relevance of these findings.

### Behavioural data

As one could have anticipated, our MI training improved the quality of motoric imagery abilities expressed as the coupling between actual motor execution of a classical clinical task, the TUG task, and MI times for the same task (MIQI index): in the experimental group, the value of the index became lower, indicating a training-induced improvement of the ability to efficiently evoke gait motor representations. In contrast with previous reports on post-TKA acute phase^16,17^, where the MI training only improved lower limbs muscular strength, our MI improvement generalized to real walking, with better recovery of motor performance for actual gait, as measured by the TUG test. The difference with previous studies is perhaps less than surprising, because in these experiments patients were instructed only to imagine maximal voluntary isometric contractions of the quadriceps^16,17^ or simple knee extension-flexion^18^. On the contrary, we specifically asked our patients to imagine complex gait behaviours, like walking in ecological settings: this, if anything, shows that a specific form of mental motor training should be used to achieve the desired results for complex outcomes like measures of gait. The results on the Hand-Walking Task (HWT), that was used as a control task, are in line with this hypothesis: the improvement in motor control after MI training was specific for the trained movements and did not generalize different body segments movements, suggesting that movement-specific protocols are needed in each patient population to achieved the desired outcome.

To summarize, these findings are in keeping with previous studies suggesting the efficacy of mental practice on motor performance both in healthy subjects and in patients with disorders of different origins^13,19,20^ showing for the first time that complex behaviours, like those entailed by the TUG test, are amenable to mental motor training. These results are also particularly important from the clinical point of view; indeed, the TUG test requires both static and dynamic balance for its correct execution, since it does not simply measure the speed of walking but the overall gait functional mobility and the ability to transfer, walk, and change direction. Finally, the American Geriatric Society recommends that TUG should be used as a routine screening test for falls^21^, something in line with our results on the risk of falls (see below).

### Clinical & kinematic data

Our findings on pain perception confirm the clinical potentials of MI on pain, as previously reported by Moseley *et al*.^22^, who showed an effect on pain and disability in the complex regional pain syndrome type I and in phantom limb pain. MI may represent a valuable tool in mitigating pain, as we previously found in rizarthrosis^13^.

On the other hand, we could not find an effect of the MI training on functional scales, like the FIM or the Barthel Index; these scales are, by design, not enough sensitive for detecting functional gait motor recovery. For example, the FIM scale has only two items addressing locomotion; similarly, the Barthel Index has only one item related to general gait. In future studies, it might be particularly interesting to use validated clinical scales more focused on functional gait abilities. With regard to changes in the range of motion from T1 to T2, an interesting pattern deserves attention: while no treatment effect was seen in the passive ROM, a computerized gait analysis during gait execution revealed a group specific difference when the ROM was measured during active movements. This finding, together with what observed with the TUG test, suggest that MI has an impact in promoting amelioration in tasks that require subjects to be actively engaged.

### Falls, near falls and fear of falls

Finally, our data indicate that MI training may also have a long-term effect on what can be considered the outcome measure with most clinical relevance: falls or near falls (e.g., stumbles, momentary losses of balance). These were significantly reduced in the experimental group, on average, 2 years after surgery. While we cannot offer a deterministic explanation of why this should be so, we can offer some educated guesses. The cognitive intervention provided by MI may have helped our patients in focusing their attention on the planning phase and on the different sub-components of the complex movements of gait; this may have enhanced the performance during attention-demanding and challenging situations, thereby contributing to real-world fall avoidance (see for example^23^). Given the relevance of this observation, the relatively small number of patients under examination and the particular distribution of adverse events like falls, some caution is in order here. However, this and previous similar evidence are encouraging on the potentials of MI training in this respect. Conversely, we did not find any effect of MI training on the fear of fall nor a correlation with the occurrence of falls or near falls. This suggests that the more limited occurrence of falls/near falls in the experimental group was not simply due to a generic greater caution when walking.

The different trend observed for falls and fear of falls might be explained by the fact that fear of falling is not always a consequence of a previous fall. Indeed, some studies revealed the multidimensional nature of the fear of falling, whereby not only actual falls but also general characteristics, physical, and psychosocial variables act as risk factors^24^.

For example, Murphy *et al*.^25^ demonstrated that, in community-living older women, fear of falling is associated with a combination of predisposing factors such as visual impairment, a sedentary lifestyle, and no available emotional support^25^.

Moreover, Allali and colleagues demonstrated that fear of falls is a falls’ predictor only in individuals with postural instability/gait difficulty^26^.

Therefore, experiencing a reduced number of falls/near falls does not necessarily imply a change in fear of falls.

### Study limitations

Before concluding, we need to address the limitations of this study. For example, we did not take into account the muscular strength as a possible outcome measure, although previous studies demonstrated that, for example, quadriceps strength correlates with functional measures, like the TUG test^27^.

Another possible limitation is related to the timing of our short-term follow-up (16 days after surgery), when several intervening frailties might lead to reduced gait speed or problems in postural changes (such as poli-pharmacological treatments, need of special assistance for continence problems, fear of falling). However, these factors were balanced between the control and experimental groups, and cannot readily explain our results.

It is worth mentioning that, given the tendency to make hospital stays shorter and shorter after surgery, having information in the time-window considered here has some practical importance.

### Conclusion and clinical implications

Motor imagery training, added to conventional physical rehabilitation, can improve gait and limit new falls in the long term in patients with recent TKA in the post-surgery acute phase, when practical limitations (e.g. post-surgery immobilization or reduced physical strength) and increased perceived pain can limit the applicability of direct physical training. Because of its simplicity, the absence of any collateral risk, and the limited costs, motor imagery training should be incorporated into the routine rehabilitation of these patients.

In particular, our findings indicate that two daily sessions of 30’ are sufficient to speed up gait recovery in the acute post-surgery phase. Our experience suggests the value of combining MI exercises supported by visual stimuli (e.g., in-motion videos) with internally-generated MI practice (e.g., mentally evoking the kinesthetic sensations associated with particular movements). Indeed, gait is a complex behavior, made of several components, some more bodily centered and based on kinesthetic motoric information, others related to the monitoring of the external world, other to the integration af these two aspects. In this perspective, internally guided MI may serve better to efficiently regain bodily centered motor awareness; on the other hand, visually-guided imagery may be useful in trying to connect elementary motor skills to the real-life needs of walking in a free environment. Of course these mechanistic explanations on the relationship of different aspects of our protocol with their overall efficacy are for the moment working hypotheses that one may test by comparing different forms of gait motor imagery, one more centered on kinesthetic motoric sensations, another one more related to the monitoring of the outside world while imagining the task of walking and the different phases of rehabilitation. These are questions that remain open for future experimentation.

As far as the clinical implications of this work, these have different time frames and relevance: the short-term ones, related to the performance at the TUG task, the active range of motion and the pain, are worth of notice as they speak to an alleviation of the consequences of surgery immediately after the treatment: one could only hope that some of these effects has a longer-term duration or could be kept alive through continuing home-based training. A specific longer-term follow-up study is needed to demonstrate that. In any event, the clinical effect that looks more promising is definitively the longer-term one related to the risk of falls: given the implications of falls for the quality of life, this finding is worth a deeper investigation on larger and more diverse populations.

## Materials and Methods

### Study design and measures collected

The study involved a longitudinal collection of behavioural, clinical and kinematic measures in a cohort of 48 patients with gonarthrosis who underwent surgical treatment for their condition (total knee arthroplasty). All participants received the standard post-surgical physical therapy in the rehabilitation unit. Patients started the rehabilitation protocol about 6 days after the surgery when they were able to walk a few meters with the help of two crutches. They performed physical exercise with a physiotherapist 6 days per week, 70 minutes per day. Exercises were focused on recovering optimal passive and active knee range of motion, strength and neuromuscular control of the lower limb, while regaining full autonomy in postural changes, ambulation with two crutches and in activities of daily living. For more details, see Negrini *et al*.^28^.

The study included four time-points. A clinical-functional evaluation of gait motor abilities and pain (see below for further details) was performed the day before surgery (baseline, T0), at the entrance in the rehabilitation unit (T1: about 6 days after surgery, when our experimental/placebo training started), and at the discharge (T2: about 16 days after surgery). Patients were then evaluated by means of telephonic interviews, investigating (i) their fear of falls and (iii) the number of falls/near falls experienced in the last 12 months before the telephone interview (T3, about 2 years after surgery).

At T0, patients also underwent a detailed neuropsychological evaluation to assess their cognitive functioning. We administered the following neuropsychological tests: Mini Mental State Examination (MMSE^29^), for the global cognitive functioning; Frontal Assessment Battery, for global frontal/executive functioning (FAB^30^); the Raven Colored Progressive Matrices^31^, for logical reasoning; the short-story recall for short-term verbal memory abilities^32^; the Digit Span Backward test, for verbal working memory abilities (DSB^33^) and the Corsi test^33^, for visuo-spatial memory abilities. Finally, we asked all participants to complete the Vividness of Movement Imagery Questionnaire (VMIQ^34^) to measure self-reported general motor imagery abilities.

Overall, our participants showed a cognitive profile within the normal range; we observed only a few isolated pathological scores (see Supplementary Table S1, scores marked with an asterisk). More importantly, there were not significant differences between the scores obtained by the two groups in any of the listed test.

**Figure 4:**
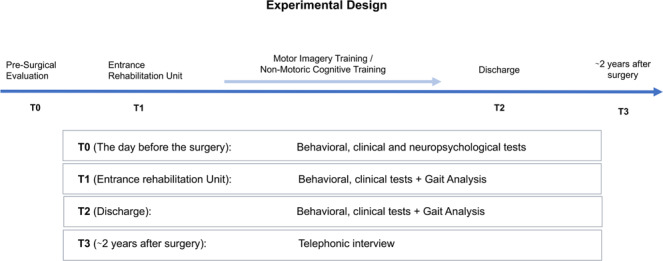
Graphical representation of the experiment design.

At T1 and T2, all patients were submitted to a computerized gait-analysis (see paragraph 2.3.3) and scores obtained in functional scales like the Functional Independence Scale (FIM^35^) and the Barthel Index^36^ were also collected, together with the passive range of motion (ROM). See Fig. 4 for a graphical illustration of the experimental design.

### Participants

We recruited 48 patients (20 M/28 F, mean age: 66.4 (±7.7) years), submitted to total knee arthroplasty. The study protocol was approved by the local Institutional Review Board (Comitato Etico ASL Città di Milano; prot. L3020). All experimental protocols were carried out in accordance of Helsinki Declaration (1964). Informed consent was obtained from all the patients.

Inclusion criteria were: (1) age comprised between 45 and 80 years old, (2) being enrolled in the local residential rehabilitation program. Exclusion criteria were: (1) presence of neurologic or neurodegenerative diseases, (2) on-going psychopharmacological treatments.

In order to rule out any possible confounding effect of pre-existing differences between the experimental and the control group for demographic, but also for clinical and cognitive characteristics at T0, we performed a series of statistical tests (independent samples Student’s t tests when variables were normally distributed – Shapiro Wilk’s p-value > 0.05; Mann-Whitney U’s tests when variables were not normally distributed – Shapiro Wilk’s p-value < 0.05; chi-square test for dichotomic variables). As indicated in Table 3, the two groups were matched for all the tested variables (p-values > 0.05). The two groups were also matched for the pharmacological pain treatment after the surgical procedure (paracetamol: experimental group (n:13), control group (n:12); NSAIDs: experimental group (n:9), control group (n:10)).

**Table 3:** Descriptive statistics of demographical, clinical, cognitive and behavioural variables at baseline split by experimental and control group.

Variable	Mean (SD) or Frequency (%)		Statistics
	Experimental Group	Control Group	
Age (years)	66.2 (8.0)	66.6 (7.5)	Student’s t(46) = −0.2; p = 0.9
Sex	13M/11F	7M/17F	Chi-square(1) = 3.1; p = 0.08
Education (years)	9.7 (3.0)	10.6 (6.7)	Mann-Whitney U = 256; p = 0.5
BMI	28.4 (6.6)	31.4(6.7)	Mann-Whitney U = 197; p = 0.3
Surgery side	12R/12L	14R/12L	Chi-square(1) = 0.3; p = 0.5
Gait motor abilities (TUG, T0, s)	12.7 (5.8)	14.0 (4.5)	Mann-Whitney U = 284; p = 0.08
Gait MI abilities (MIQI TUG)	0.3 (0.2)	0.3 (0.2)	Mann-Whitney U = 279; p = 0.9
Hand motor abilities (HandWalking, T0, s)	10.6 (7.9)	11.4 (5.4)	Mann-Whitney U = 243; p = 0.4
Hand MI abilities (MIQI HandWalking)	0.2 (0.2)	0.2 (0.2)	Mann-Whitney U = 282; p = 0.9
Pain (T0) – Affected Leg	3.0 (3.4)	2.6 (3.4)	Mann-Whitney U = 284; p = 0.9
Pain (T0) – Non Affected Leg	0.6 (1.7)	0.7 (1.7)	Mann-Whitney U = 283; p = 0.9
Handedness (Oldfield test)	83.3 (14.0)	80.8 (18.2)	Mann-Whitney U = 201; p = 0.9
General cognitive functioning (MMSE)	28.8 (1.6)	29.1 (1.1)	Mann-Whitney U = 282; p = 0.9
Executive functioning (FAB)	16.1 (1.9)	16.1 (2.1)	Mann-Whitney U = 276; p = 0.8

### Clinical, behavioral experimental and gait analysis measures

#### Timed up and go (TUG) and “Hand-Walking” tasks

To measure gait functional abilities in each group, all participants performed the Timed Up and Go test (TUG^14^) in the version developed by Beauchet and collaborators^37^, which also includes a measure of MI gait abilities. Participants were seated and instructed to walk 3 meters, turn around, walk back to the chair and sit down saying, “stop”. Times were recorded with a stopwatch to the nearest 0.01 s: the stopwatch was started on the command “ready–set–go” and stopped when the participant sat down and said, “stop”. In the imagined condition, the participants sat in the chair and were instructed to imagine performing the TUG (iTUG) with their eyes closed in a first person kinesthetic perspective, and to say, “stop” when they were done. The TUG and the iTUG were both performed twice. In order to assess MI abilities, we calculated an index of the quality of MI based on motor chronometry (Motor Imagery Quality Index – MIQI, differences between iTUG time and TUG time), according to the following formula: (iTUG time − TUG time)/[(iTUG time + TUG time)/2]. The same index was called respectively “delta time” in Beauchet *et al*.^37^ and “chronometry ability” in Allali *et al*.^38^. The lower the MIQI score, the smaller is the difference between executed and imagined movement times, the better the MI abilities.

As a control task, we used a novel hand-motor task that we call “Hand-Walking task” (HWT) in which the participants used the index and middle fingers of their dominant right hand to simulate “walking” along an S-shaped path drawn on a sheet of paper (28 × 40 cm, see also Sacheli *et al*., 2018). The executed and the imagined Hand-Walking tasks were performed twice. A MIQI for the HWT was calculated in the same way as for the TUG. The HWT was added to investigate whether the MI training generally improves motor control abilities or it is specific for the trained movements (here, gait patterns). For all tasks, the MI version of the task was performed immediately after actual gait execution: this was done to facilitate the recalling of kinesthetic sensations. Task order (TUG / Hand-Walking) was counterbalanced across participants.

#### Evaluation of pain

Patients rated how much pain they felt at rest in each knee (both the joint to be operated on and the healthy one) using a visual analogue scale (VAS) ranging from 0 (“no pain at all”) to 10 (“the worst pain I could imagine”).

#### Gait analysis

For gait analysis, a Helen Hayes marker set of 22 retro-reflective passive markers was used and a Davis biomechanical model was utilized during data acquisition and processing^39^. Patients were asked to walk at a self-paced speed with a walking aid along a 13-meter walkway five times. An optoelectronic system (SMART-D, BTS Bioengineering, Milan, Italy) with eight infrared cameras (sampling rate 100 Hz) was used for spatiotemporal and kinematic data acquisition during each walking section. Marker trajectories were recorded, reconstructed, and processed by SMART-D Analyzer software (BTS Bioengineering). The gait parameters collected were: (i) spatiotemporal variables — stride length (m), gait speed (m/s), gait cadence (steps/min), stance phase, swing phase, and double support phase; stance, swing and double support phases were normalized as a percentage of the gait cycle and (ii) kinematic parameters (in degrees): knee range of motion (ROM). These measures were collected in 36 patients (18 patients of the experimental group and 18 patients of the control group).

#### Number of falls/near falls and fear of falls

As long-term outcomes, we investigated by means of telephonic interviews (i) the number of falls and near falls experienced by the participants in the previous 12 months with respect to the time of the call; (ii) the fear of fall by administering the activities-specific balance confidence (ABC) scale^40^, where they were asked to indicate their level of self-confidence in a number of possible daily behaviors that require balance, ranging from 0 (no confidence) to 100 (completely confident). These measures were collected in 34 patients (17 patients of the experimental group and 17 patients of the control group).

### Cognitive treatment

The assignation to the experimental or to the control group was made by using a block randomization procedure: this permitted to balance age, gender and education between groups. The modalities of the training procedure did not differ between the experimental and placebo group, while the content of the training session was diversified. Both trainings engaged the participants for 30 minutes twice a day, lasting on average eleven days.

#### Motor imagery treatment

The motor imagery training program involved different tasks; there were two alternative versions (A and B) of the training, one for each daily session.

Version A

(i) *Motor imagination of standing and gait in different ecological settings supported by visual stimuli*. Patients watched naturalistic 30” videos of a path through a park: they were asked to imagine themselves walking along the path as if the camera were “their own eyes”. After the video, participants were instructed to repeat the movement imagination with their eyes closed for other 30”.

(ii) *Motor imitation of actors walking and standing in different ecological settings supported by visual stimuli*. Patients watched naturalistic 30” videos of an actor walking through a park and were instructed to imagine imitating the actor. This task combined both motor imagery and action observation: the act of watching the walking actor was not passive; rather, participants were explicitly instructed to “imagine imitating” the actor’s behaviour.

Version B

(i) *Motor imagination of standing and gait supported by audio stimuli*. Patients were asked to imagine themselves walking and standing in different conditions, guided by the voice of the experimenter (e.g., “Now imagine you’re standing in front of a street. Focus the attention on your body, there are no crutches or signs of surgery. Now imagine starting to walk with your right foot: the heel of the foot rises and the knee begins to bend as the body weight moves to the left leg. The right leg is now raised and moves forward, stretching out […]”).

(ii) *Motor imagination of knee flexion-extension supported by audio stimuli*. Patients were asked to imagine themselves flexing and extending their knee (e.g., now close your eyes and focus the attention on your operated leg. Feel your foot touching the ground and slowly shift your attention from the foot to the ankle, and then to the knee. Focus on your knee, on how it is bent so that your foot rests on the ground. Now imagine you feel your muscles stretching out; the knee slowly changes its position and following the thrust of the thigh upwards opens until it completely extends […]”).

#### Placebo control treatment

The placebo control treatment was based on the enhancement of non-motoric cognitive functions (visual memory tasks, words recall tests, letter cancellation tasks, figural fluency tasks). There were two alternative versions of the training (A and B), one for each daily session. The difficulty of the exercises was adapted on each patient’s cognitive level and it increases during the eleven days of training. The goal was to find a level that was challenging, but not frustrating for the participant.

Version A

(i) *Five-point test*. Participants were instructed to connect with a pen some (or all) of the points of a matrix, with the indication of not repeating a configuration already drawn. More or less complicated matrices were available depending on the possible symmetries found in the drawing (e.g., square with point in the center vs. four points in a row and one above not centered).

(ii) *Letter/Number cancellation tasks*. Participants were asked to search for and mark one or more specific symbols on an A3 sheet with lines of letters, or random letters and numbers.

Version B

(i) *Visual memory task*. The experimenter placed on the table pairs of cards, covered, representing the same object or animal. Participants were invited to turn two cards at a time. If they found the same figure they were left uncovered and continued, if they were different instead all the cards were covered and it was restarted from the beginning. The goal was to discover all the cards on the table. The number of pairs of cards varied according to the patient’s performance (range: from 4 to 10 pairs).

(ii) *Words recall test*. The experimenter read a list of 10 words for 3 times consecutively to the patient. A distracting exercise was then performed (usually simple additions) for about 1 minute. Subsequently, the patient was asked to recall as many words as possible from the list read previously. Some words presented a phonetic assonance and others a semantic association between them to make possible the use of mnemonic strategies.

### Data analysis

The descriptive statistics of raw data (mean and SD) at each time-point, for each group, are indicated in Table 1. For each collected measure, we first performed a series of within-group comparisons, in order to check the expected post-surgical clinical improvement. For each comparison, we also calculated the minimal detectable change (MDC). This measure is particularly important in clinical terms, since it represents the minimum amount of change in patients’ scores that ensures that the change isn’t the result of measurement error.

#### Timed up and go (TUG) and hand-walking task (HWT)

In order to test whether the MI training did speed up gait recovery during the post-surgical course in the experimental group with respect to the control group, we first calculated a measure that we named “*delta TUG*” for each repetition of the test (TUG time in T2 – TUG time in T1); this measure represented the level of improvement of post-surgical gait abilities during the rehabilitation. Since the TUG test was performed twice, we compared the delta TUG of the two groups by means of general linear mixed-effects model, with random intercept; the factor group was modelled as fixed factor.

The same approach was then used for the HWT, to investigate the specific effects of the training, and to the MIQI scores associated with the TUG and the HWT, in order to test the specific improvement of MI abilities for the patients assigned to the experimental group.

#### Evaluation of pain

The aim of this analysis was to assess between-group differences in the perceived pain associated with the operated knee during the post-surgical recovery. We first calculated a measure that we named “*delta VAS*” (perceived pain operated knee in T2 – perceived pain operated knee in T1); this measure represented the level of change of post-surgical pain during the rehabilitation phase.

Since the VAS measure was collected only once, we compared the delta VAS of the two groups by means of an independent samples t-test; we used a non-parametric independent samples t-test (Mann-Whitney test) because these data violated the assumption of homoscedasticity (Levene test’s p-value < 0.05). The same approach was applied for the perceived pain associated of the non-affected knee.

#### Gait analysis measures

The aim of this analysis was to assess between-group differences in any of the spatial and temporal parameters collected during the gait analysis. For each parameter, we first calculated a delta measure (parameter collected in T2 – parameter collected in T1); this measure represented the level of change of post-surgical gait parameters assessed with the computerized gait analysis.

We compared the delta values of the two groups by means of a series of parametric/non-parametric independent samples t-test, on the basis of the data distribution.

#### Falls, near falls and fear of falls

The dependent variable was the number of falls and near falls among TKA patients reported at T3 (on average at 2 years after ending of rehabilitation). Due to the characteristics of the data on falls, the answer of “no fall experience” was coded as 0 (zero count). Given the structure of the dependent variable, we used a Poisson regression model; we considered the reported number of falls/near falls as the dependent variable of the model and the factor “Group (Experimental/Control) as the independent one. The time interval between the surgical procedure and the time of the interview was treated in the model as a covariate.

In order to to assess between-group differences in the domain of the fear of falls, we first calculated a measure that we named “*delta ABC*” (ABC T3 – ABC T0). We compared the delta ABC of the two groups by means of independent samples t-test.

#### Functional scales and passive range of motion

In order to to assess between-group differences in the scores of functional scores or in the passive range of motion, we first calculated a delta measure for each scale and for the passive range of motion (T3 –T0). These delta scores were compared by means of independent samples t-test.

## Supplementary information

Supplementary Table S1.

Supplementary Table S2.
