# Supplementary material for: Motor imagery training speeds up gait recovery and decreases the risk of falls in patients submitted to total knee arthroplasty
Source: Sci Rep. 2020 Jun 2;10:8917. doi: 10.1038/s41598-020-65820-5 (PMC7265300; doi:10.1038/s41598-020-65820-5)
Supplement: Supplementary file 1 — Supplementary Table S1. [file 41598_2020_65820_MOESM1_ESM.docx]

**Supplementary online materials**

**Motor imagery training speeds up gait recovery and decreases the risk of falls**

**in patients submitted to total knee arthroplasty**

Laura Zapparoli^1,2^, Lucia Maria Sacheli^1,2^, Silvia Seghezzi^1,3^, Matteo Preti^2^, Elena Stucovitz^2^, Francesco Negrini^2^, Catia Pelosi^2^, Nicola Ursino^2^, Giuseppe Banfi^2,4^ and Eraldo Paulesu^1,2^

^1^ Psychology Department and NeuroMI – Milan Center for Neuroscience, University of Milano-Bicocca, Milan, Italy

^2^ IRCCS Istituto Ortopedico Galeazzi, Milan, Italy

^3^ PhD Program in Neuroscience, School of Medicine and Surgery, University of Milan-Bicocca, Milan, Italy

^4^ University Vita e Salute San Raffaele, Milan, Italy

**Corresponding authors:**

Laura Zapparoli & Eraldo Paulesu

Psychology Department, University of Milano-Bicocca

Milan, Italy

E-mail: [laura.zapparoli@unimib](mailto:laura.zapparoli@unimib), [eraldo.paulesu@unimib.it](mailto:eraldo.paulesu@unimib.it)

**Table S1.** Descriptive statistics of the scores of the gait-specific items (mean (SD)) before (T1) and after (T2) the intervention, for both the experimental and the control group.

| **Item** | **Experimental Group** | | | **Control Group** | | | **Between-group comparison**  **(Delta values)** |
| --- | --- | --- | --- | --- | --- | --- | --- |
|  | **T1** | **T2** | **Statistics** | **T1** | **T2** | **Statistics** | **Statistics** |
| *Barthel - Gait* | 6.6 (3.3) | 15 (0) | Student's t(7)=7.2, p<0.001 | 6.9 (5.22) | 15 (0) | Student's t(7)=4.4, p=0.003 | Mann Whitney’s U=30, p=0.87 |
| *Barthel - Stairs* | 0 (0) | 9.5 (0.9) | Wilcoxon W<0.001, p=0.01 | 0 (0) | 9.25 (1.03) | Wilcoxon W<0.001, p=0.01 | Mann Whitney’s U=28, p=0.65 |
| *FIM - Gait* | 4.1 (0.8) | 6.5 (0.9) | Student's t(7)=6.3, p<0.001 | 3.75 (1.75) | 6.5 (0.9) | Wilcoxon W<0.001, p=0.01 | Mann Whitney’s U=30.5, p=0.91 |
| *FIM - Stairs* | 1 (0) | 6.25 (0.7) | Student's t(7)=21, p<0.001 | 1 (0) | 6.25 (0.7) | Student's t(7)=21, p<0.001 | Mann Whitney’s U=32, p>0.99 |
